# The Value of Whole-Tumor Histogram and Texture Analysis Using Intravoxel Incoherent Motion in Differentiating Pathologic Subtypes of Locally Advanced Gastric Cancer

**DOI:** 10.3389/fonc.2022.821586

**Published:** 2022-02-09

**Authors:** Huan-Huan Li, Bo Sun, Cong Tan, Rong Li, Cai-Xia Fu, Robert Grimm, Hui Zhu, Wei-jun Peng

**Affiliations:** ^1^ Department of Radiology, Fudan University Shanghai Cancer Center, Shanghai, China; ^2^ Department of Gastric Surgery, Fudan University Shanghai Cancer Center, Shanghai, China; ^3^ Department of Pathology, Fudan University Shanghai Cancer Center, Shanghai, China; ^4^ MR Applications Development, Siemens Shenzhen Magnetic Resonance Ltd, Shenzhen, China; ^5^ MR Applications Development, Siemens Healthcare, Erlangen, Germany

**Keywords:** gastric cancer, IVIM, pathological characterization, texture analysis, whole-tumor analysis

## Abstract

**Purpose:**

To determine if whole-tumor histogram and texture analyses using intravoxel incoherent motion (IVIM) parameters values could differentiate the pathologic characteristics of locally advanced gastric cancer.

**Methods:**

Eighty patients with histologically confirmed locally advanced gastric cancer who received surgery in our institution were retrospectively enrolled into our study between April 2017 and December 2018. Patients were excluded if they had lesions with the smallest diameter < 5 mm and severe image artifacts. MR scanning included IVIM sequences (9 b values, 0, 20, 40, 60, 100, 150,200, 500, and 800 s/mm^2^) used in all patients before treatment. Whole tumors were segmented by manually drawing the lesion contours on each slice of the diffusion-weighted imaging (DWI) images (with b=800). Histogram and texture metrics for IVIM parameters values and apparent diffusion coefficient (ADC) values were measured based on whole-tumor volume analyses. Then, all 24 extracted metrics were compared between well, moderately, and poorly differentiated tumors, and between different Lauren classifications, signet-ring cell carcinomas, and other poorly cohesive carcinomas using univariate analyses. Multivariate logistic analyses and multicollinear tests were used to identify independent influencing factors from the significant variables of the univariate analyses to distinguish tumor differentiation and Lauren classifications. ROC curve analyses were performed to evaluate the diagnostic performance of these independent influencing factors for determining tumor differentiation and Lauren classifications and identifying signet-ring cell carcinomas. The interobserver agreement was also conducted between the two observers for image quality evaluations and parameter metric measurements.

**Results:**

For diagnosing tumor differentiation, the ADC_median_, pure diffusion coefficient median (Dslow_median_), and pure diffusion coefficient entropy (Dslow_entropy_) showed the greatest AUCs: 0.937, 0.948, and 0.850, respectively, and no differences were found between the three metrics, P>0.05). The 95th percentile perfusion factor (FP _P95th_) was the best metric to distinguish diffuse-type GCs vs. intestinal/mixed (AUC=0.896). The ROC curve to distinguish signet-ring cell carcinomas from other poorly cohesive carcinomas showed that the Dslow_median_ had AUC of 0.738. For interobserver reliability, image quality evaluations showed excellent agreement (interclass correlation coefficient [ICC]=0.85); metrics measurements of all parameters indicated good to excellent agreement (ICC=0.65-0.89), except for the Dfast metric, which showed moderate agreement (ICC=0.41-0.60).

**Conclusions:**

The whole-tumor histogram and texture analyses of the IVIM parameters based on the biexponential model provided a non-invasive method to discriminate pathologic tumor subtypes preoperatively in patients with locally advanced gastric cancer. The metric FP _P95th_ derived from IVIM performed better in determining Lauren classifications than the mono-exponential model.

## Introduction

In China, gastric cancer (GC) has the second-highest cancer burden and the third most common cause of cancer-related deaths (with an age-standardized rate of incidence of 20.6 per 100,000 people, an age-standardized rate of mortality 15.9 per 100,000 population), and most patients are diagnosed at advanced disease stages (1). Patients presenting with locally advanced gastric cancer (LAGC) encounter problems associated with precise diagnoses and personalized treatment plans (2, 3) since tumor differentiation, Lauren classifications, and the presence of signet-ring cells can influence prognoses and treatment determinations (4–6). Lauren classifications are convenient and easy to implement and have good interobserver agreement (7). A recent study showed that the LAGC Lauren types correlated with perioperative chemotherapy responses (6). Endoscopic biopsies are invasive procedures prone to sampling errors due to the high heterogeneity of GCs; thus, the histopathology of tumor biopsies might not be consistent with those of whole-tumor resections (8, 9). Therefore, non-invasive imaging methods that could reliably predict the histopathologic characteristics of tumors could be useful.

Texture analysis is the method by which MRI and computed tomography (CT) radiologic data are processed using special software to extract texture features, which can quantitatively reflect pathologic information (10). CT remains the primary imaging modality in GC management owing to its relatively high accuracy rates and convenience (11). Several previous studies have shown that texture analyses from CT were useful for predicting GC prognoses and evaluating responses to neoadjuvant therapy (12–14), and some other studies have reported that preoperative CT texture analysis from omentum or primary tumors can help predict occult peritoneal metastases of advanced gastric cancers (15, 16). CT exposes patients to ionizing radiation and produces poor soft-tissue contrast. However, with technologic advancements, MRI temporal and spatial resolution has improved significantly, and its accuracy for assessing GC is similar to that of CT (17). Furthermore, MRI has good soft-tissue contrast and allows for repeated examinations owing to its non-ionizing radiation. It can also yield functional imaging features and has become a promising imaging technique for GC (11). However, applying texture analyses to MRI for GC diagnostics is less common (18); A few studies have found that apparent diffusion coefficient (ADC) first-order statistical metrics might be able to predict GC nodal status and are associated with perineural and vascular invasion (19, 20). Another study exploratory showed ADC histogram data from mono-exponential could reflect different histologic grades GC (21).

Based on diffusion-weighted imaging (DWI), Le Bihan et al. (22) proposed using intravoxel incoherent motion (IVIM) model to distinguish tissue perfusion and diffusion. IVIM is performed using bi-exponential curve fitting with multiple b-values and quantitative measurements with IVIM-derived parameters. Currently, this technique has been used for tumor grading, prognostic determinations, treatment monitoring, and distinguishing benign from malignant tumors (23, 24). However, it is rarer to use IVIM parameter texture analyses to evaluate GC in clinical research (25). Therefore, in this study, we aimed to investigate if IVIM whole-tumor histogram and texture analyses could be used to predict the pathologic features of LAGC.

## Materials and Methods

### Patients

**Figure 1:**
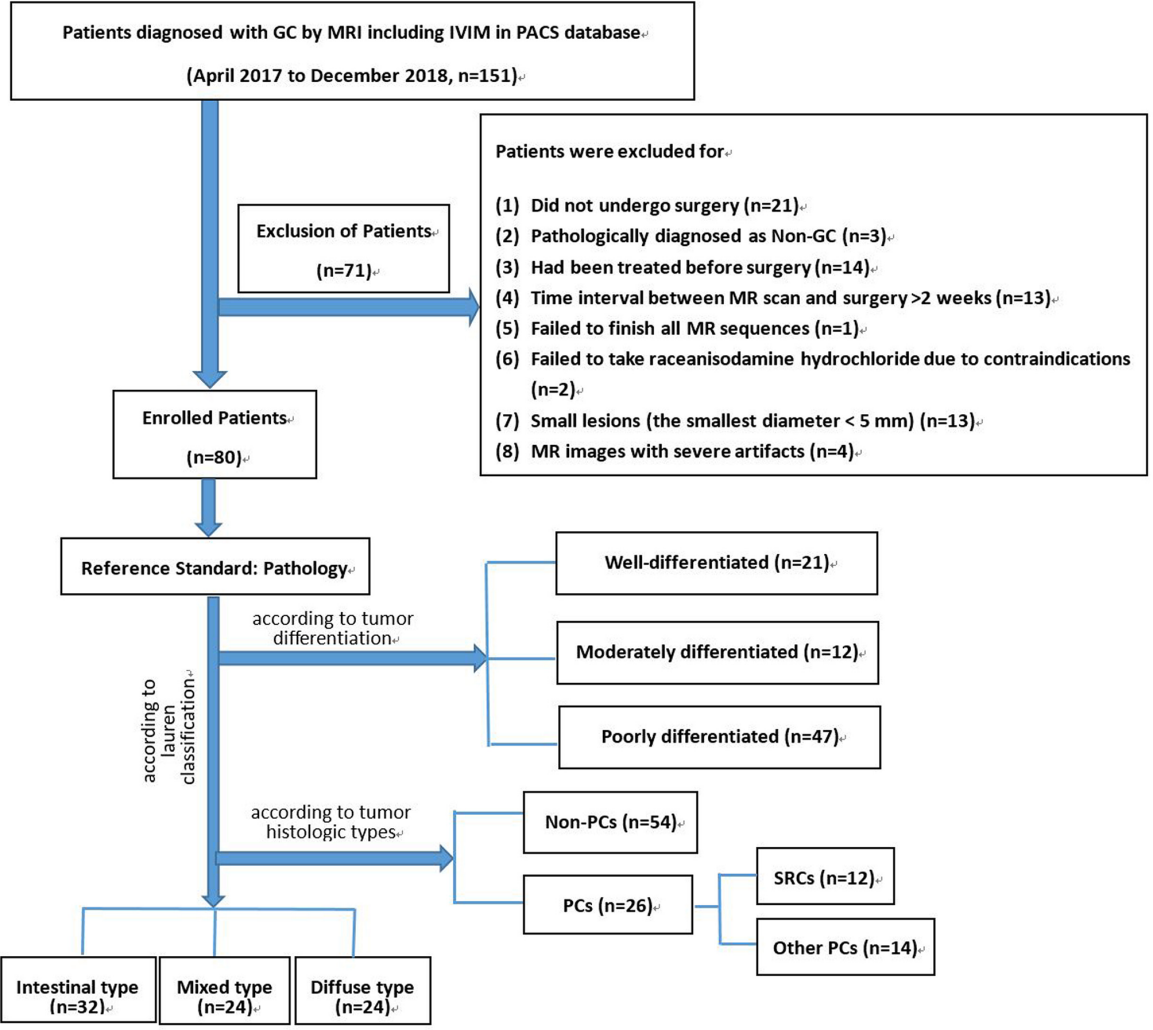
Flow chart of our study population. GC, gastric cancer; IVIM, intravoxel incoherent motion; PACS, picture archiving and communication system; SRC, signet-ring cell carcinoma; PC, poorly cohesive carcinoma

The study was a retrospective, cross-sectional observational analysis. From April 2017 to December 2018, a total of 80 patients with LAGC were included. The study protocol was approved by our institutional review board. The inclusion criteria were as follows: a) patients who underwent surgery in our institution; b) patients with histologically confirmed GC; and c) patients who underwent preoperative MRI with IVIM sequences. Seventy-one patients were excluded: 1) patients had been treated before surgical interventions; 2) the time interval between MRI and surgery was ≥ 2 weeks; 3) patients failed to finish all MRI scan sequences; 4) had contraindications to raceanisodamine hydrochloride; 5) had small lesions (the smallest diameter < 5 mm); and 6) MRI images had severe artifacts (see **Figure 1**).

### MRI Examinations

Examinations were performed on a 3-Tesla MR scanner (MAGNETOM Skyra; Siemens Healthcare, Erlangen, Germany). We used an integrated body coil for excitation, with a dedicated 32-channel spine coil and an 18-channel body coil for signal reception. To reduce artifacts from intestinal peristalsis, raceanisodamine hydrochloride (10 mg; Minsheng Pharmaceuticals, Hangzhou, China) was administered to patients intramuscularly 5–10 min before the MR examinations, unless contraindicated. Patients fasted for more than 6 h to ensure the stomach was empty and then drank 500-800 ml water immediately before the MR examination to distend the stomach.

IVIM acquisitions were obtained before administering contrast medium, using a single-shot echo-planar imaging sequence (SS-EPI) with diffusion gradients of 9 b-values (0, 20, 40, 60, 100, 150, 200, 500, 800 s/mm2), and a 3D-diagonal diffusion mode was applied.

Other routine sequences included axial T1-weighted imaging (T1WI; in-phase and out-of-phase), axial T2-weighted imaging (T2WI), and axial contrast-enhanced imaging using volumetric interpolated breath-hold examinations (VIBEs). The detailed scanning parameters are shown in **Table 1**.

**Table 1:** IVIM and routine sequence parameters.

Parameter	IVIM Sequence	T1-weighted IP and OP Sequence	T2-weighted Sequence	T1-weighted VIBE Sequence
Repetition time (msec)/echo time (msec)	5700/54	120/1.4 and 2.74	3500/83	3.9/1.89
b values (sec/mm^2^)	0, 20, 40, 60, 100, 150, 200, 500, 800	—	—	—
Slice thickness (mm)	5	3.5	4	3.5
Slice gap (mm)	1	0.7	0.8	0.7
Acquisition matrix	128 × 128	320 × 240	256 × 256	320 × 240
Field of view (mm^2^)	380 × 380	380 × 310	380 × 380	380 × 310
Acquisition time	3min and 9s	26s	3min and 15s	17s×3(30,60,90s)
Flip angle value	Excitation 90° Refocusing 180°	70°	91°	9°
		—	—	—
Parallel imaging factor	2	—	—	—
Echo-planar imaging factor	115	—	—	—
No. of signals acquired	Sequentially according to b values: 2, 2, 2, 2, 3, 4, 4, 4, 6.	2	4	1

IP, in-phase; IVIM, intravoxel incoherent motion; OP, out-of-phase; and VIBE, volumetric interpolated breath-hold examination.

The acquisition planes are all axial imaging.

### MR Image and Data Analyses

MR image quality was rated by two radiologists (with 5 and 10 years of abdominal diagnosis experience, respectively) according to a five-point Likert-type scale (1 = very poor, 2 = poor, 3 = moderate, 4 = good, and 5 = excellent), with a higher score indicating a better assessment.

The IVIM parameters (Dslow, pure molecular-based diffusion coefficient; Dfast, pseudo-diffusion coefficient; and FP, pseudo-diffusion perfusion factor) and ADC values were calculated using the Body Diffusion Toolbox (prototype software, Siemens Healthcare, Erlangen, Germany) based on all acquired b-values. Then, the IVIM parameter maps, ADC maps, and DWI with b=800 were imported into prototypic MR Multiparametric Analysis software (Siemens Healthcare, Erlangen, Germany). The two radiologists drew regions of interest (ROIs) manually on DWI images (with b=800), using contrast-enhanced images as references. ROIs were drawn along cancer lesion margins (excluding the areas with the highest and lowest signals to avoid partial-volume effects). After ROIs were drawn around whole tumors, based on IVIM parameters and ADC values, five histogram-derived texture metrics (median, P95th, P5th, skewness, kurtosis) and one second-order texture metric (entropy) were generated. Skewness and kurtosis reflect histogram shapes and measure parameter distribution asymmetries, and entropy represents variations in the parameter distributions of interest (26).

To evaluate interobserver agreement for image quality and data measurements, image quality scores and data analysis results of the two radiologists were tested.

### Histopathologic Examinations

Histopathologic analyses were performed by a pathologist (with 10 years of clinical experience) who was blinded to IVIM parameter measurements. Tissue sections were stained with a hematoxylin and eosin (HE) stain according to routine procedures. Tumor differentiation, Lauren classifications, and the identification of poorly cohesive carcinomas (PCs) and signet-ring cell carcinomas (SRCs) on histology were evaluated and recorded according to the World Health Organization (WHO) classification (27) and Chinese national standard for GC diagnosis and treatment (28).

### Statistical Analyses

The Shapiro-Wilk test and QQ plots were used to check the normality of the continuous variable distributions. The two-sample t-test or Mann-Whitney test was used to detect the metrics differences between the SRCs and other PCs. We used the one-way ANOVA and Kruskal-Wallis test to compare these metrics among the three differentiation degrees and the three Lauren classifications. Since many variables existed, logistic regression and multicollinear tests were adopted to screen out independent influencing factors for tumor differentiation and the Lauren classification. Then, screened variables were subjected to receiver operating characteristic (ROC) curve analyses, and the results were guaranteed to have practical significance due to the elimination of confounding factors. The ROC curve diagnostic accuracy was interpreted as low (area under the curve [AUC]=0.50-0.70), moderate (AUC=0.70-0.90), or high (AUC>0.90) (29). The interobserver agreement between the two radiologists was evaluated with the interclass correlation coefficient (ICC) test, which was interpreted as having a poor (ICC=0.00-0.20), fair (ICC=0.21-0.40), moderate (ICC=0.41-0.60), good 0.61-0.80, and 0.81-1.00, excellent correlations (30).

A P-value of <0.05 was considered statistically significant. ROC curve parameter comparisons were assessed using MedCalc software version 19.6.0, and other statistical analyses were performed using SPSS software version 23.0.

## Results

### The Study Population and Interobserver Agreement

Eighty patients were finally included in the study. The average age of the patients (58 men and 22 women) was 60.7 years (range, 28–89 years). Tumors were located in the gastric cardia and fundus in 27 cases, the gastric body in 24 cases, and the gastric antrum in 29 cases. Most tumors in the cardia and fundus involved in the study were not confined to cardia or fundus, and there was no clear demarcation between the two areas. Therefore, we did not distinguish them among anatomical subtypes. For more detailed patient characteristics, see **Table 2**.

**Table 2:** Baseline and demographic data in 80 patients.

Characteristics	Value
Patient sex	
No. of men	58 (72.5%)
No. of women	22 (27.5%)
Age (y)	60.7 (28-89)
Tumor location	
Cardia and fundus	27 (33.7%)
Gastric body	24 (30.0%)
Gastric antrum	29 (36.3%)
Tumor volume (cm^3^)	43.2 (3.4-200.7)
Tumor smallest diameter (mm)	16.9 (6.0-65.0)
Pathologic findings	
T staging	
T2	12 (15.0%)
T3	36 (45.0%)
T4a	32 (40.0%)
N staging	
N0	19 (23.75%)
N1	20 (25%)
N2	23 (28.75%)
N3	18 (22.5%)
Tumor differentiation	
Well-differentiated	21 (26.2%)
Moderately differentiated	12 (15.0%)
Poorly differentiated	47 (58.8%)
Lauren classification	
Intestinal type	32 (40.0%)
Mixed type	24 (30.0%)
Diffuse type	24 (30.0%)
Histologic types	
Non-PCs	54 (67.5%)
PCs	26 (32.5%)
SRCs	12 (15.0%)
Other PCs	14 (17.5%)

Continuous data are shown as means, with ranges in brackets. Categorical data are expressed as numbers of patients, with percentages in brackets.

T staging, tumor staging; N staging, lymph node staging; SRCs, signet-ring cell carcinomas; PC, poorly cohesive carcinoma.

The interobserver agreement for image quality evaluations was excellent (the ICC was 0.85), so we adopted results from the first reader. Of the recruited patients, IVIM images from 26 patients were rated as 5 points (excellent), 32 patients as 4 points (good), 18 patients as 3 points (moderate), and 4 patients as 2 points (poor). We observed the IVIM image artifacts of 4 patients with 2 points, but the artifacts were on the abdominal wall and did not affect the gastric lesion conspicuity. The interobserver agreement for ADC, Dslow, and FP measurements was good to excellent (ICC=0.65-0.89), and the agreement for Dfast measurements was moderate (ICC=0.41-0.60; see **Table 3**).

**Table 3:** Interobserver agreement for parameters measurements assessed by the interclass correlation coefficient.

Variable metrics	ADC	Dslow	Dfast	FP
**Median**	0.82 [0.74,0.88]	0.81 [0.72,0.87]	0.60 [0.43,0.72]	0.88 [0.82,0.92]
**P5th**	0.85 [0.77,0.90]	0.88 [0.82,0.92]	0.58 [0.42,0.71]	0.89 [0.83,0.93]
**P95th**	0.78 [0.68,0.85]	0.74 [0.62,0.82]	0.49 [0.30,0.64]	0.84 [0.76,0.89]
**Skewness**	0.73 [0.61,0.82]	0.74 [0.63,0.83]	0.44 [0.24,0.60]	0.76 [0.65,0.84]
**Kurtosis**	0.79 [0.69,0.86]	0.88 [0.82,0.92]	0.45 [0.26,0.61]	0.84 [0.77,0.90]
**Entropy**	0.65 [0.51,0.76]	0.72 [0.60,0.81]	0.41 [0.21,0.58]	0.78 [0.67,0.85]

Data are interobserver correlation coefficients, with 95% confidence intervals in brackets.

ADC, apparent diffusion coefficient; Dslow, pure molecular-based diffusion coefﬁcient; Dfast, pseudo-diffusion coefficient; FP, pseudo-diffusion factor given as a percentage; P5th, 5th percentile; and P95th, 95th percentile.

### Histograms and Texture Metrics of IVIM Parameters for Tumor Differentiation

**Figure 2:**
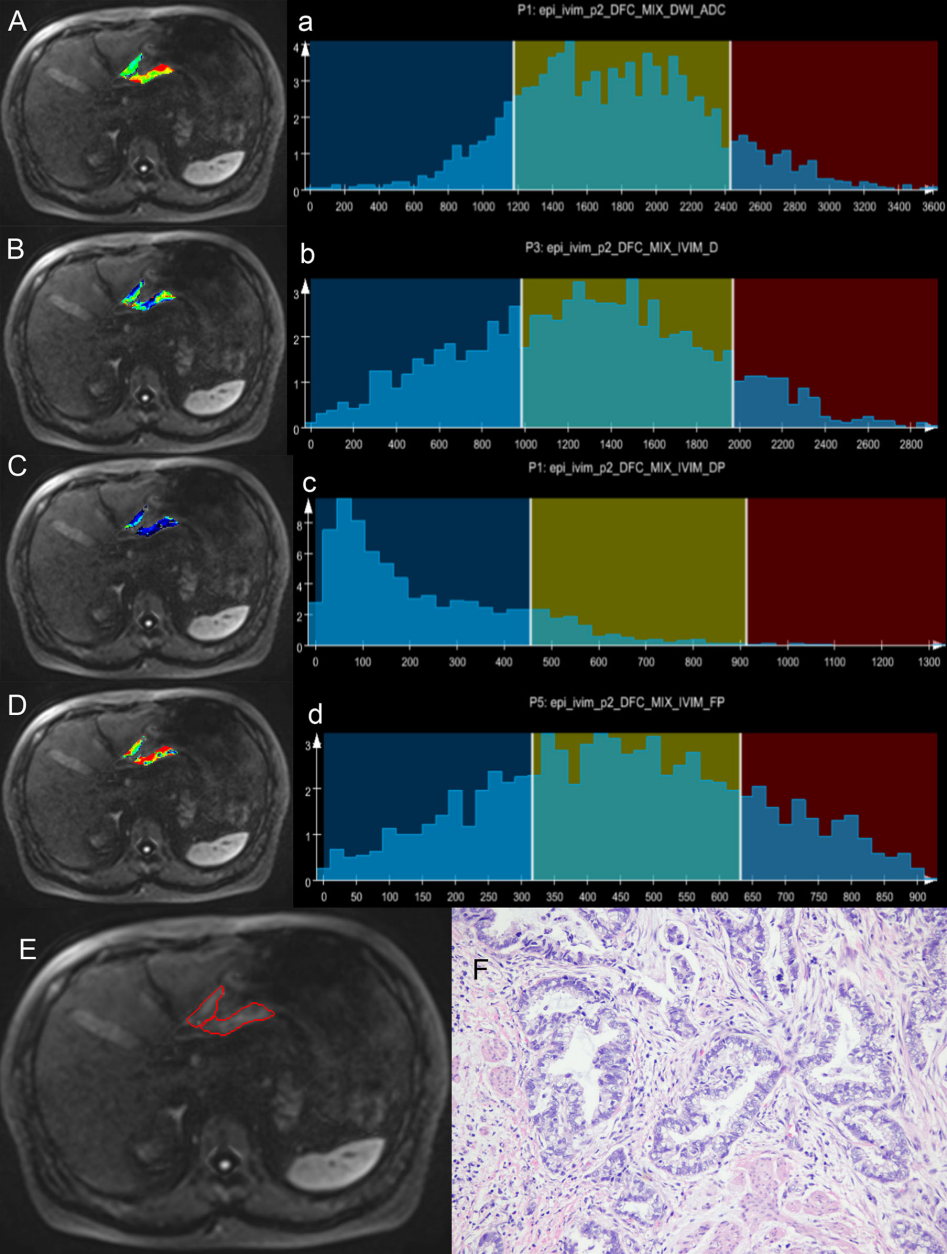
A case of gastric antrum cancer. The colored regions in **(A–D)** represent the lesion parameters maps; **(a–d)** show histogram parameter distributions for the whole tumor (ADC, Dslow, Dfast, and FP presenting sequentially). **(E)** Shows the contour of the region of interest (ROI). **(F)** A photomicrograph of an HE stained tissue section demonstrating a moderately differentiated adenocarcinoma.

**Figure 3:**
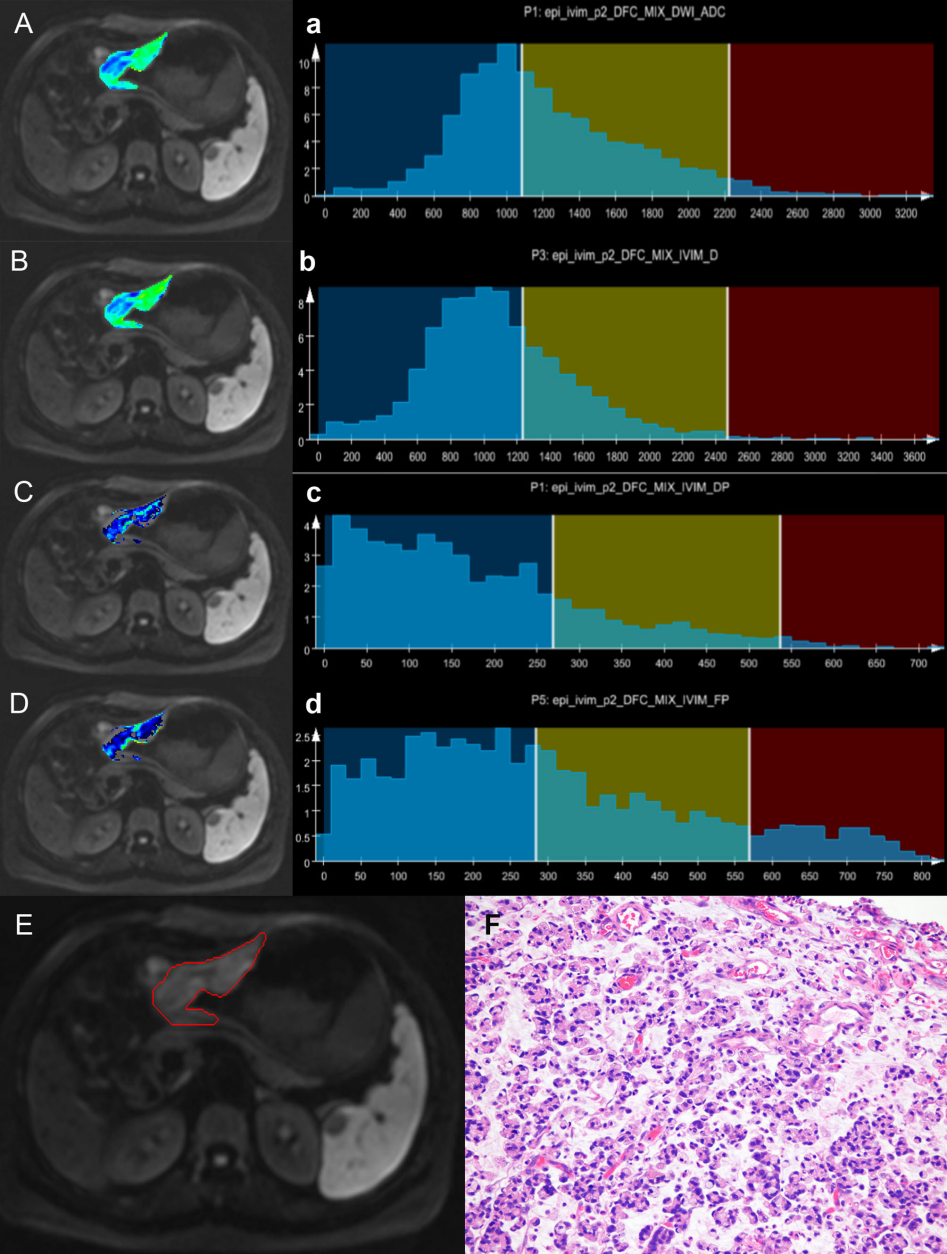
A case of gastric body cancer. The colored regions in **(A–D)** represent the lesion parameters maps; **(a–d)** show histogram parameter distributions for the whole tumor (ADC, Dslow, Dfast, and FP presenting sequentially). **(E)** Shows the contour of the region of interest (ROI). **(F)** A photomicrograph of an HE stained tissue section demonstrating a poorly differentiated signet-ring cell carcinoma.

The median, P5th, and P95th values of the ADC, Dslow, and FP parameters were higher in the well/moderately differentiated GCs compared with those in the poorly differentiated GCs(all total P-values <0.05, except the P-value for Dslow_P5th_). The skewness, kurtosis, and entropy values of the ADC, Dslow, and FP parameters were lower in the well/moderately differentiated GCs compared with those in the poorly differentiated GCs (all total P-values <0.05, except P-value for ADC_kurtosis_). For the Dfast parameter, none of the metric values were different among the three differentiation degrees (P >0.05); see **Table E1** (online). **Table E1** also shows the paired comparisons among the three differentiation degrees. Representative cases from the two groups are shown in **Figures 2**, **3**.

**Figure 4:**
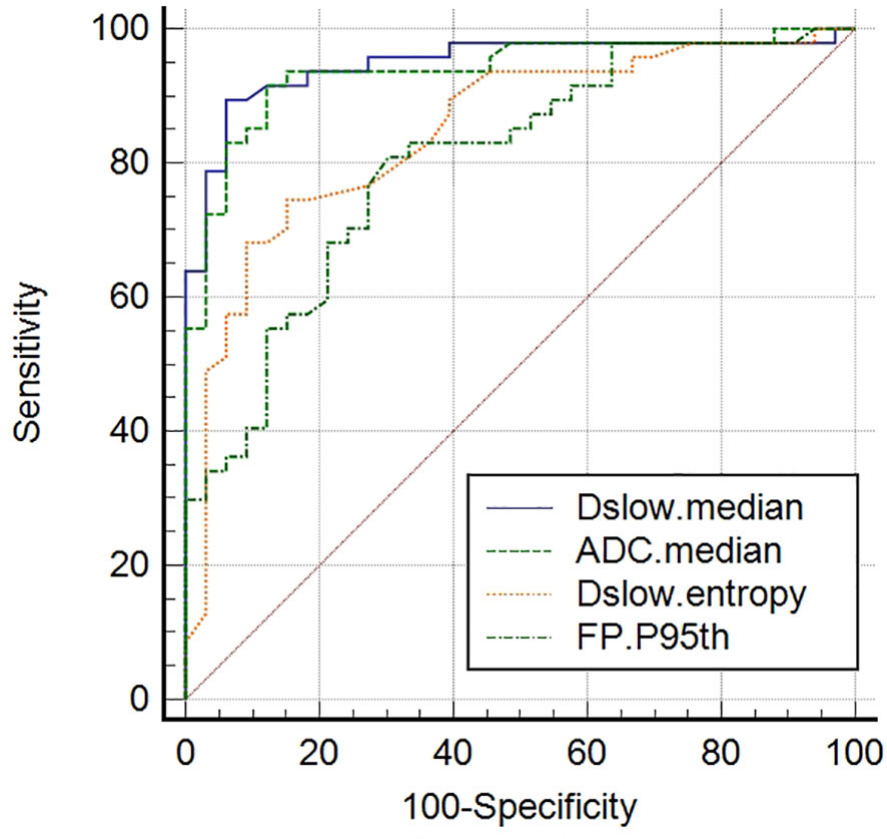
Receiver operating characteristic (ROC) curves of screened independent influencing factors that could distinguish poorly differentiated from well/moderately differentiated GCs. ADC_median_, Dslow_median_, and Dslow_entropy_ show good diagnostic performance, with AUCs of 0.937, 0.948, and 0.850, respectively. Comparisons among the four metrics determined that these values were significantly different (P <0.05), although no differences (P >0.05) were detected when paired comparisons among the ADC_median_, Dslow_median_, and Dslow_entropy_ values were performed.

ADC_median_, Dslow_median_, Dslow_entropy_, and FP_P95th_ were screened out as independent influencing factors for tumor differentiation (the cutoff values for distinguishing the well/moderately differentiated and poorly differentiated GCs were 1601.50×10^-6^mm^2^/s, 1356.50×10^-6^mm^2^/s, 3.16, and 63.15%, respectively). In ROC curve analyses, we found Dslow_median_ had the largest AUC of 0.948 (P<0.001) with an accuracy of 91.3%, sensitivity of 89.4%, and specificity of 93.9%; however, these values were not statistically different from those of ADC_median_ and Dslow_entropy_ (P>0.05; see **Table 4** and **Figure 4**).

**Table 4:** The diagnostic performance of the independent influencing factors for the well/moderately differentiated vs. poorly differentiated GC.

Variable metrics	Cutoff	Accuracy	Sensitivity	Specificity	PPV	NPV	AUC	P-value
ADC_median_	1601.50^a^	90.0%	91.5%	87.9%	91.5%	87.9%	0.937 [0.874-0.985]	<0.001
Dslow_median_	1356.50^a^	91.3%	89.4%	93.9%	95.5%	86.1%	0.948 [0.860-0.979]	<0.001
Dslow_entropy_	3.16	78.8%	74.5%	84.8%	87.5%	70.0%	0.850 [0.749-0.918]	<0.001
FP_P95th_	63.15%	76.3%	80.9%	69.7%	79.2%	71.9%	0.803 [0.699-0.883]	<0.001

Numbers in brackets are 95% confidence intervals (95% CI).

PPV, Positive predictive value; NPV, Negative predictive value; AUC, area under the curve; ADC, apparent diffusion coefficient; Dslow, pure molecular-based diffusion coefﬁcient; FP, pseudo-diffusion factor given as a percentage; and P95th, 95th percentile.

a, 10^-6^mm^2^/s.

No differences (P >0.05) were detected by paired comparisons between ADC_median_, Dslow_median_, and Dslow_entropy_.

### Histograms and Texture Metrics of IVIM Parameters for Lauren Classifications

Except for Dfast metrics, Dslow_P5th_, and FP_entropy_, other metrics were statistically different among the three Lauren classifications. The median, P5th, and P95th values of the ADC, Dslow, and FP parameters were higher in the intestinal/mixed types compared with those in the diffuse-types (all total P-values <0.05, except the P-value for Dslow_P5th_). The skewness, kurtosis, and entropy values of the ADC, Dslow, and FP parameters were lower in the intestinal/mixed types compared with those in the diffuse-types(all total P-values <0.05, except the P-value for FP_entropy_); see **Table E2** (online). **Table E2** also shows the paired comparisons among the different Lauren classification groups.

**Figure 5:**
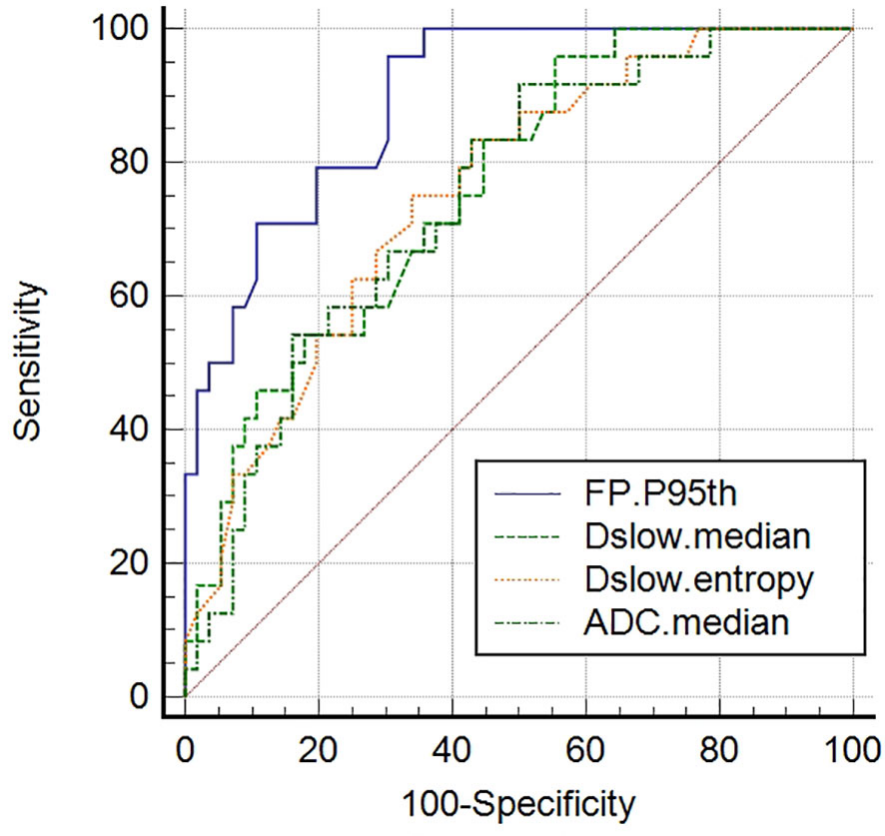
Receiver operating characteristic (ROC) curves of screened independent influencing factors that could distinguish diffuse-type GCs from intestinal/mixed-type GCs. FP_P95th_ had the largest AUC of 0.896. Comparisons among the four metrics determined that these values were significantly different (P <0.05), although no differences (P >0.05) were detected when paired comparisons among the ADC_median_, Dslow_median_, and Dslow_entropy_ values were performed.

We further screened the independent influencing factors for the different Lauren classification groups, including ADC_median_, Dslow_median_, Dslow_entropy_, and FP_P95th_. We found that FP _P95th_ had the largest AUC of 0.896 (P<0.001) with an accuracy of 77.5%, sensitivity of 95.8%, and specificity of 69.6%, with no statistical difference between the other three metrics (see **Table 5** and **Figure 5**).

**Table 5:** The diagnostic performance of the independent influencing factors for the intestinal/mixed vs. diffuse-type GC.

Variable metrics	Cutoff	Accuracy	Sensitivity	Specificity	PPV	NPV	AUC	P value
ADC_median_	1626.50^a^	62.5%	91.7%	50.0%	44.0%	93.3%	0.747 [0.637-0.838]	<0.001
Dslow_median_	1437.50^a^	60.0%	95.8%	44.6%	42.6%	96.2%	0.762 [0.653-0.850]	<0.001
Dslow_entropy_	3.16	68.8%	75.0%	66.1%	48.6%	86.0%	0.755 [0.646-0.844]	<0.001
FP_P95th_	61.15%	77.5%	95.8%	69.6%	57.5%	97.5%	0.896 [0.829-0.963]	<0.001

Numbers in brackets are 95% confidence intervals.

PPV, Positive predictive value; NPV, Negative predictive value; AUC, area under the curve; ADC, apparent diffusion coefficient; Dslow, pure molecular-based diffusion coefﬁcient; FP, pseudo-diffusion factor given as a percentage; and P95th, 95th percentile.

a = 10^-6^mm^2^/s.

No differences (P >0.05) were detected by paired comparisons between ADC_median_, Dslow_median_, and Dslow_entropy_.

### Histograms and Texture Metrics of IVIM Parameters for Differentiating Signet-Ring Cell Carcinomas from the Other Poorly Cohesive Types

**Figure 6:**
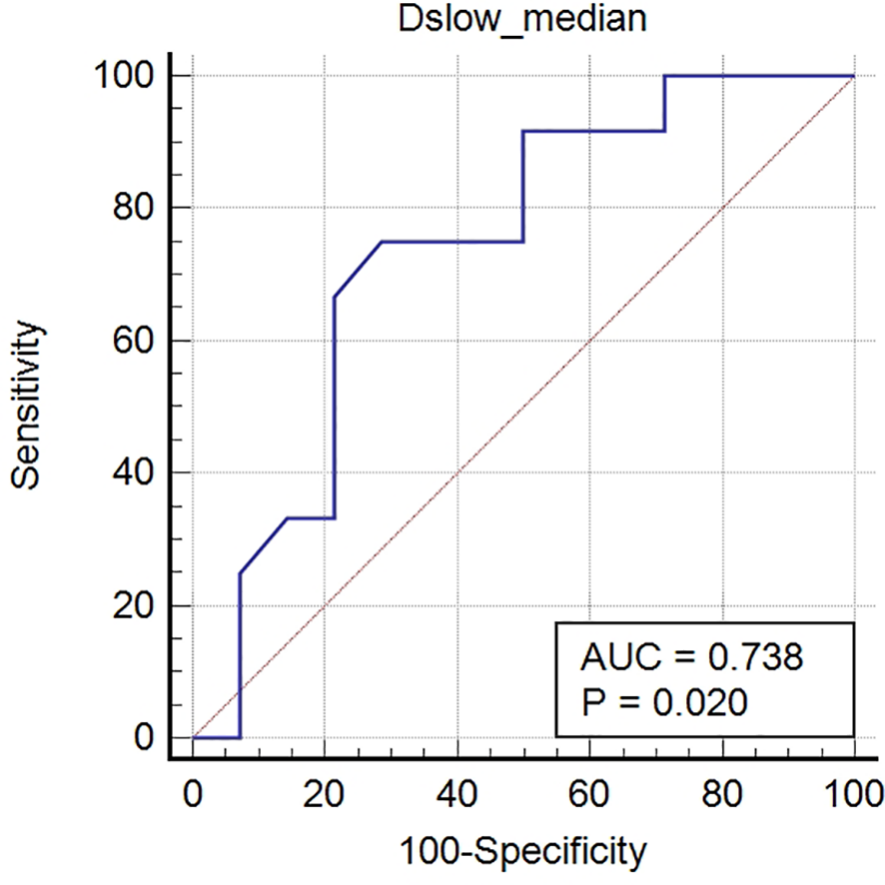
Receiver operating characteristic (ROC) curves of Dslow_median_ for distinguishing signet-ring cell carcinomas from other poorly cohesive carcinomas. The AUC of Dslow_median_ was 0.738.

All SRC and other PC metrics were compared using univariate analyses. The Dslow_median_ value was the only metric that showed statistical differences between the two groups, with the SRC values being less than those of the other PC types (P <0.05); see **Table E3** (online). In the ROC analyses, the Dslow_median_ had an AUC of 0.738, with an accuracy of 70.4%, sensitivity of 75.0%, and specificity of 71.4%; see **Table 6** and **Figure 6**.

**Table 6:** The diagnostic performance of Dslow_median_ for discriminating SRCs and other poorly cohesive carcinomas.

Variable metrics	Cutoff	Accuracy	Sensitivity	Specificity	PPV	NPV	AUC	P value
Dslow_median_	1041.00^a^	70.4%	75.0%	71.4%	69.2%	76.9%	0.738 [0.537-0.939]	0.020

Numbers in brackets are 95% confidence intervals.

PPV, Positive predictive value; NPV, Negative predictive value; AUC, area under the curve SRCs, Signet-ring cell carcinomas; ADC, apparent diffusion coefficient; and Dslow, pure molecular-based diffusion coefﬁcient.

a =10^-6^mm^2^/s.

No differences (P >0.05) were detected by paired comparisons between ADC_median_ and Dslow_median_.

## Discussion

Our research focused on analyzing histogram and texture characteristics of IVIM parameters for LAGC with different pathological subtypes. IVIM imaging has the advantage of simultaneously obtaining diffusion and perfusion information without a co-registration processing step and the administration of contrast media (31). Many studies have reported its application in rectal tumors, concluding that IVIM parameters reflected histologic changes after treatment. Studies have also shown that IVIM parameters are associated with tumor differentiation and clinical staging (32–35). However, clinical studies looking at IVIM in patients with GC have not been commonly reported, although a few animal studies have examined chemotherapeutic efficacies (36, 37). An initial study reported the use of IVIM parameters to assess GC histotypes, but single-slice ROIs rather than whole-tumor volumetric measurements were used, and histograms and texture analyses were not applied (38). SRC is a rare type of adenocarcinoma characterized by signet-ring cells that secrete large amounts of mucin and displace the nucleus to the cell periphery (39). This cancer type is insensitive to chemoradiotherapy and has a poor prognosis in advanced stages (40). In 2010, the WHO classification defined PC as isolated or small aggregates of discohesive carcinoma cells with an infiltrative pattern, including SRCs and other cell types (27, 41).. There have only been a few previously published MRI studies on SRC, especially with respect to the difference between SRCs and other PC types. In this study, we explored the value of whole-tumor histogram and texture features for IVIM parameters in identifying GC differentiation, Lauren types, and SRC carcinomas according to ROC curve analysis.

For tumor differentiation, we found that the diffusion parameter metrics, ADC_median_, Dslow_median_, and Dslow_entropy_, showed better diagnostic performance as independent influencing factors for distinguishing poorly differentiated from the well/moderately differentiated GCs (see **Figure 4**). The ADC_median_ and Dslow_median_ values of poorly differentiated tumors were significantly lower than those of the well/moderately differentiated tumors. These findings were similar to a previously reported study, which showed that restricted water motion in malignant tumors was associated with tumor differentiation (21). In addition, the Dslow_entropy_ value of poorly differentiated tumors was higher than that of well/moderately differentiated tumors, suggesting that poorly differentiated tumors have more radiologic heterogeneity/variability. As a perfusion parameter, the diagnostic performance of FP_P95th_ for tumor differentiation was not too bad, although the AUC was smaller than that of ADC_median_, Dslow_median_, and Dslow_entropy_. In our study, the FP_P95th_ in the poorly differentiated tumor was significantly lower than that of well/moderately differentiated tumors, which could indicate that lower FP values are related to the hypoperfusion of blood caused by fewer normal glandular structures in poorly differentiated tumors (35).

Lauren classifications can reflect the biological aggressiveness of GC, in which diffuse-type GCs display a diffusely invasive growth pattern with a worse prognosis than intestinal/mixed-type (5). We found the perfusion parameter metric, FP_P95th_, had the best diagnostic efficiency for discriminating diffuse-type GCs from intestinal/mixed-type GCs (see **Figure 5**). The FP_P95th_ values of the diffuse-type GCs were significantly lower than those of the intestinal/mixed-type GCs, which suggests that the diffuse-type GC FP histograms were less frequent at the high end of the FP values compared with intestinal/mixed-type GC FP histograms. We previously showed that diffuse-type GCs have a less glandular appearance than intestinal/mixed GCs (5), which suggests that the lower FP values of diffuse-type GCs might be due to the hypoperfusion of blood caused by fewer normal glandular structures. Diffuse-type GCs also had higher Dslow_entropy_ values than intestinal/mixed-type GCs, indicating that the diffuse-type GCs have more radiologic heterogeneity/variability on the Dslow maps. A previous study reported that the ADC values from a mono-exponential model correlated with the GC Lauren classifications (42). Our research indicated that the FP_P95th_ metric performed better than ADC in determining Lauren classifications, demonstrating the advantage of using IVIM multi-parametric analyses from the biexponential model over using parametric analyses from the mono-exponential model.

Our research found that the Dslow_median_ values of the SRCs were lower than those of other PC types, providing moderate diagnostic efficacies for distinguishing the two types (see **Figure 6**). The parameter Dslow from the biexponential model, which separates perfusion effects, might reflect the true diffusion state within lesions better than ADC from the mono-exponential model (23, 31). In our study, Dslow_median_ value was the only metric that showed statistical differences between the SRCs and other PC types, and which had greater AUC values than ADC_median_ in determining tumor differentiation and Lauren classifications. However, the differences were not statistically significant and could have been caused by the relatively small sample size of some groups and the difficulty of including additional b-values in clinical practice.

Our study used whole-tumor analysis for IVIM parameter metric measurements. This whole-tumor analysis reduced intratumoral heterogeneity influences on the measurements and provided more reproducible and reliable data than single-slice ROI analyses (33, 43). In this research, all parameter measurements had good or excellent interobserver reproducibility except for Dfast, which showed greater measurement susceptibility with moderate agreement.

There were several limitations to this study. First, early GC lesions are small and susceptible to motion artifacts and partial-volume averaging; thus, our research included only patients with LAGC (the smallest diameter of lesions≥ 5 mm). Second, we used water as the negative contrast agent to fill the stomach cavity; however, gas-liquid levels sometimes appeared near the lesions, leading to susceptibility artifacts. Future prospective studies will develop a more robust acquisition method and a special gastric filling contrast agent to minimize susceptibility artifacts. Third, there were slightly fewer cases in the diffuse group, but according to the EPV (events per variable) principle proposed by Vittinghoff et al. (44), the sample size was sufficient for the analyses. In addition, the sample sizes of patients with SRC and other PC were small, so for these individuals, we only performed univariate analyses. Given that SRC is less common, our univariate analytic results have some significance.

Despite these limitations, our study showed the novel advantages of IVIM multi-parameter histogram and texture analyses for GC research based on the biexponential model. Moreover, IVIM provided an additional perfusion parameter, FP, which demonstrated greater potential for determining Lauren classifications than ADC from the mono-exponential model.

## Data Availability Statement

The raw data supporting the conclusions of this article will be made available by the authors, without undue reservation.

## Ethics Statement

The study is approved by Fudan University Shanghai Cancer Center Institutional Review Board. Written informed consent for participation was not required for this study in accordance with the national legislation and the institutional requirements.

## Author Contributions

W-JP and HZ conceived this idea and managed the research project, H-HL and BS designed this study, MRI data acquisition was performed by H-HL and RL, Statistical analysis was performed by H-HL and HZ, BS was contributed to clinical data interpretation, CT performed pathological analysis. C-XF and RG provided software analysis methods. All authors contributed to the article and approved the submitted version.

## Funding

This work was supported by the National Natural Science Foundation of China (grant number 81902436) and the Science and Technology Commission of Shanghai Municipality (grant number STCSM18411953000).

## Conflict of Interest

C-XF was employed by Siemens Shenzhen Magnetic Resonance Ltd. RG was employed by Siemens Healthcare.

The remaining authors declare that the research was conducted without any commercial or financial relationships that could be construed as a potential conflict of interest.

## Publisher’s Note

All claims expressed in this article are solely those of the authors and do not necessarily represent those of their affiliated organizations, or those of the publisher, the editors and the reviewers. Any product that may be evaluated in this article, or claim that may be made by its manufacturer, is not guaranteed or endorsed by the publisher.

## Abbreviations

ADC, apparent diffusion coefficient; AUC, area under the curve; Dfast, pseudo-diffusion coefficient; DWI, diffusion-weighted imaging; Dslow, pure molecular-based diffusion coefficient; FP, pseudo-diffusion perfusion factor; GC, gastric cancer; IVIM, intravoxel Incoherent Motion; ICC, interclass correlation coefficient; LAGC, locally advanced gastric cancer; P5th, 5th percentile; P95th, 95th percentile; PC, poorly cohesive carcinoma; ROC, receiver operating characteristic; SRC, signet-ring cell carcinoma.
