# Supplementary material for: The Value of Whole-Tumor Histogram and Texture Analysis Using Intravoxel Incoherent Motion in Differentiating Pathologic Subtypes of Locally Advanced Gastric Cancer
Source: Front Oncol. 2022 Feb 9;12:821586. doi: 10.3389/fonc.2022.821586 (PMC8864172; doi:10.3389/fonc.2022.821586)
Supplement: Supplementary file 1 [file Table_1.docx]

**Table E1 Univariate analyses of IVIM parameters and ADC histogram/texture metrics** **among the three differentiation degrees**

| **Metrics** | **Median** | **P5th** | **P95th** | **Skewness** | **Kurtosis** | **Entropy** |
| --- | --- | --- | --- | --- | --- | --- |
| **Parameters** |  |  |  |  |  |  |
| **ADC** |  |  |  |  |  |  |
| Well | 1733.50^a^ | 1117.50^a^ | 2762.64^a^ | 0.16 | 0.20 | 2.92 |
| 95% CI | [1581.50-1842.50] | [908.50-1243.50] | [2610.66-2914.63] | [0.01-0.32] | [0.01-0.39] | [2.85-3.00] |
| Moderately | 1733.67^a^ | 1050.58^a^ | 2550.29^a^ | 0.38 | 0.36 | 2.88 |
| 95% CI | [1603.14-1864.20] | [918.97-1182.19] | [2398.36-2702.23] | [0.11-0.66] | [-0.70 to 0.78] | [2.78-2.97] |
| Poorly | 1403.50^a^ | 808.28^a^ | 2208.48^a^ | 0.46 | 0.48 | 3.02 |
| 95% CI | [1328.50-1475.50] | [741.09-875.47] | [2138.79-2278.16] | [0.32-0.59] | [0.21-0.61] | [2.97-3.06] |
| P-value |  |  |  |  |  |  |
| Total | ＜0.001 | ＜0.001 | ＜0.001 | 0.041 | 0.126 | 0.01 |
| Well vs Moderately | 0.879 | 1.000 | 0.030 | 0.167 | 0.417 | 0.420 |
| Well vs Poorly | ＜0.001 | ＜0.001 | ＜0.001 | 0.012 | 0.052 | 0.031 |
| Moderately vs Poorly | ＜0.001 | 0.005 | ＜0.001 | 0.595 | 0.318 | 0.009 |
| **Dslow** |  |  |  |  |  |  |
| Well | 1611.53^a^ | 664.17^a^ | 2447.50^a^ | 0.08 | 0.12 | 3.06 |
| 95% CI | [1526.80-1696.27] | [537.45-790.88] | [2407.50-2649.50] | [-0.09 to 0.24] | [-0.12 to 0.35] | [2.98-3.14] |
| Moderately | 1496.63^a^ | 680.25^a^ | 2403.75^a^ | 0.16 | 0.13 | 3.05 |
| 95% CI | [1401.40-1591.85] | [553.66-806.84] | [2207.67-2599.83] | [-0.04 to 0.0.36] | [-0.21 to 0.47] | [2.94-3.16] |
| Poorly | 1207.73^a^ | 587.50^a^ | 1966.50^a^ | 0.36 | 0.62 | 3.28 |
| 95% CI | [1147.00-1278.03] | [532.50-633.50] | [1923.00-2045.50] | [0.23-0.49] | [0.37-0.76] | [3.23-3.32] |
| P-value |  |  |  |  |  |  |
| Total | ＜0.001 | 0.053 | ＜0.001 | 0.022 | 0.004 | ＜0.001 |
| Well vs Moderately | 1.000 | 0.862 | 0.874 | 0.580 | 1.000 | 0.833 |
| Well vs Poorly | ＜0.001 | 0.116 | ＜0.001 | 0.009 | 0.012 | ＜0.001 |
| Moderately vs Poorly | ＜0.001 | 0.028 | 0.001 | 0.124 | 0.071 | ＜0.001 |
| **Dfast** |  |  |  |  |  |  |
| Well | 119.50^b^ | 16.50^b^ | 484.69^b^ | 1.61 | 2.79 | 1.89 |
| 95% CI | [114.00-136.50] | [13.50-18.50] | [418.80-550.58] | [1.36-1.86] | [2.05-5.58] | [1.73-2.04] |
| Moderately | 131.88^b^ | 17.63^b^ | 409.46^b^ | 1.32 | 2.08 | 1.96 |
| 95% CI | [110.84-152.91] | [13.10-22.15] | [328.28-490.63] | [1.07-1.67] | [1.14-5.35] | [1.80-2.11] |
| Poorly | 113.50^b^ | 14.50^b^ | 424.36^b^ | 1.50 | 2.96 | 2.05 |
| 95% CI | [97.50-125.00] | [12.50-16.50] | [389.89-458.83] | [1.38-1.90] | [2.13-4.36] | [1.96-2.15] |
| P-value |  |  |  |  |  |  |
| Total | 0.262 | 0.424 | 0.141 | 0.485 | 0.841 | 0.129 |
| Well vs Moderately | 1.000 | 1.000 | 0.104 | 0.258 | 0.593 | 0.547 |
| Well vs Poorly | 0.189 | 0.229 | 0.073 | 0.816 | 0.979 | 0.049 |
| Moderately vs Poorly | 0.200 | 0.417 | 0.717 | 0.287 | 0.591 | 0.347 |
| **FP** |  |  |  |  |  |  |
| Well | 35.87% | 4.75% | 69.45% | 0.20 | -0.74 | 2.57 |
| 95% CI | [31.13%-40.60%] | [4.05%-6.35%] | [65.83%-73.08%] | [-0.01 to 0.41] | [-0.88 to -0.43] | [2.45-2.70] |
| Moderately | 30.22% | 3.70% | 64.70% | 0.42 | -0.42 | 2.75 |
| 95% CI | [24.05%-36.40%] | [3.05%-6.05%] | [58.58%-70.83%] | [0.17-0.66] | [-0.81 to -0.03] | [2.53-2.97] |
| Poorly | 22.50% | 3.35% | 56.41% | 0.74 | -0.10 | 2.81 |
| 95% CI | [20.15%-24.95%] | [2.85%-3.85%] | [53.59%-59.23%] | [0.59-0.89] | [-0.33 to 0.35] | [2.72-2.90] |
| P-value |  |  |  |  |  |  |
| Total | ＜0.001 | 0.002 | ＜0.001 | ＜0.001 | 0.002 | 0.020 |
| Well vs Moderately | 0.543 | 0.352 | 0.158 | 0.217 | 1.000 | 0.126 |
| Well vs Poorly | ＜0.001 | 0.001 | ＜0.001 | ＜0.001 | 0.003 | 0.005 |
| Moderately vs Poorly | 0.102 | 0.834 | 0.007 | 0.045 | 0.163 | 0.554 |

Data from normal distribution shown as mean, Data from non-normal distribution shown as median.

Numbers in brackets are 95% confidence intervals (95% CI).

ADC= apparent diffusion coefficient, Dslow= pure molecular-based diffusion coefﬁcient, Dfast= pseudo-diffusion coefficient, FP= pseudo-diffusion factor given as a percentage, P5th= 5th percentile, and P95th= 95th percentile.

a=10^-6^mm^2^/s, b=10^-4^mm^2^/s.

Well-differentiated (n=21), Moderately differentiated (n=12), Poorly differentiated (n=47).

**Table E2 Univariate analyses of IVIM parameters and ADC histogram/texture metrics among the three Lauren classification groups**

| **Metrics** | **Median** | **P5th** | **P95th** | **Skewness** | **Kurtosis** | **Entropy** |
| --- | --- | --- | --- | --- | --- | --- |
| **Parameters** |  |  |  |  |  |  |
| **ADC** |  |  |  |  |  |  |
| Intestinal | 1686.25^a^ | 994.75^a^ | 2392.31^a^ | 0.25 | 0.14 | 2.83 |
| 95% CI | [1595.25-1777.25] | [908.00-1125.96] | [2274.85-2509.77] | [0.12-0.39] | [0.04-0.28] | [2.77-2.95] |
| Mixed | 1519.00^a^ | 865.42^a^ | 2316.50^a^ | 0.30 | 0.12 | 2.99 |
| 95% CI | [1403.50-1623.25] | [764.79-966.05] | [2238.50-2455.00] | [0.13-0.47] | [-0.07 to 0.57] | [2.91-3.05] |
| Diffuse | 1391.08^a^ | 814.67^a^ | 2158.13^a^ | 0.59 | 0.68 | 3.07 |
| 95% CI | [1305.36-1476.81] | [723.35-905.99] | [2067.65-2248.60] | [0.39-0.80] | [0.43-0.92] | [2.95-3.10] |
| P-value |  |  |  |  |  |  |
| Total | ＜0.001 | 0.002 | 0.011 | 0.01 | 0.009 | 0.005 |
| Intestinal vs Mixed | 0.144 | 0.024 | 1.000 | 0.708 | 1.000 | 0.035 |
| Intestinal vs Diffuse | ＜0.001 | 0.003 | 0.013 | 0.004 | 0.013 | 0.008 |
| Mixed vs Diffuse | 0.177 | 1.000 | 0.060 | 0.017 | 0.040 | 1.000 |
| **Dslow** |  |  |  |  |  |  |
| Intestinal | 1466.67^a^ | 636.91^a^ | 2233.50^a^ | 0.04 | 0.17 | 3.01 |
| 95% CI | [1318.74-1551.61] | [532.61-741.20] | [1987.00-2385.50] | [-0.09 to 0.16] | [-0.01 to 0.34] | [2.91-3.08] |
| Mixed | 1317.93^a^ | 560.42^a^ | 2143.00^a^ | 0.30 | 0.28 | 3.15 |
| 95% CI | [1236.67-1399.21] | [472.65-648.18] | [2030.63-2370.50] | [0.15-0.44] | [-0.22 to 0.50] | [3.07-3.23] |
| Diffuse | 1189.10^a^ | 571.33^a^ | 2008.52^a^ | 0.50 | 0.85 | 3.25 |
| 95% CI | [1117.47-1260.74] | [488.15-654.51] | [1943.14-2073.90] | [0.32-0.68] | [0.52-1.18] | [3.18-3.31] |
| P-value |  |  |  |  |  |  |
| Total | ＜0.001 | 0.434 | 0.023 | ＜0.001 | 0.003 | ＜0.001 |
| Intestinal vs Mixed | 0.009 | 0.244 | 1.000 | 0.011 | 1.000 | 0.022 |
| Intestinal vs Diffuse | ＜0.001 | 0.317 | 0.029 | ＜0.001 | 0.002 | ＜0.001 |
| Mixed vs Diffuse | 0.032 | 0.876 | 0.096 | 0.055 | 0.046 | 0.338 |
| **Dfast** |  |  |  |  |  |  |
| Intestinal | 122.00^b^ | 16.00^b^ | 452.94^b^ | 1.41 | 2.27 | 1.97 |
| 95% CI | [115.50-135.50] | [14.00-18.00] | [407.29-498.54] | [1.22-1.73] | [1.27-4.36] | [1.85-2.10] |
| Mixed | 123.88^b^ | 14.00^b^ | 434.46^b^ | 1.57 | 2.94 | 1.92 |
| 95% CI | [104.08-143.67] | [11.50-17.00] | [373.95-494.97] | [1.31-1.84] | [1.23-4.90] | [1.81-2.02] |
| Diffuse | 116.50^b^ | 15.40^b^ | 421.50^b^ | 1.63 | 3.24 | 2.10 |
| 95% CI | [101.67-131.33] | [13.07-17.72] | [372.18-470.82] | [1.38-1.87] | [2.32-6.24] | [1.96-2.24] |
| P-value |  |  |  |  |  |  |
| Total | 0.365 | 0.536 | 0.659 | 0.574 | 0.538 | 0.133 |
| Intestinal vs Mixed | 0.333 | 0.348 | 0.598 | 0.875 | 0.974 | 0.516 |
| Intestinal vs Diffuse | 0.172 | 0.357 | 0.370 | 0.305 | 0.305 | 0.147 |
| Mixed vs Diffuse | 0.789 | 0.780 | 0.729 | 0.446 | 0.364 | 0.051 |
| **FP** |  |  |  |  |  |  |
| Intestinal | 32.31% | 4.30% | 66.10% | 0.34 | -0.67 | 2.80 |
| 95% CI | [29.06%-35.73%] | [3.95%-5.65%] | [62.81%-69.40%] | [0.18-0.49] | [-0.79 to -0.31] | [2.67-2.94] |
| Mixed | 25.28% | 3.35% | 64.29% | 0.52 | -0.38 | 2.57 |
| 95% CI | [20.25%-34.55%] | [3.05%-4.85%] | [59.53%-69.06%] | [0.25-0.78] | [-0.75 to 0.02] | [2.47-2.68] |
| Diffuse | 20.82% | 3.35% | 49.12% | 0.82 | 0.27 | 2.72 |
| 95% CI | [18.53%-23.10%] | [2.83%-3.88%] | [45.43%-52.82%] | [0.68-0.96] | [-0.17 to 0.74] | [2.56-2.88] |
| P-value |  |  |  |  |  |  |
| Total | ＜0.001 | 0.002 | ＜0.001 | 0.002 | 0.001 | 0.051 |
| Intestinal vs Mixed | 0.142 | 0.128 | 0.492 | 0.166 | 0.809 | 0.015 |
| Intestinal vs Diffuse | ＜0.001 | 0.002 | ＜0.001 | ＜0.001 | ＜0.001 | 0.383 |
| Mixed vs Diffuse | 0.049 | 0.585 | ＜0.001 | 0.032 | 0.036 | 0.138 |

Data from normal distribution shown as mean, Data from non-normal distribution shown as median.

Numbers in brackets are 95% confidence intervals (95% CI).

ADC= apparent diffusion coefficient, Dslow= pure molecular-based diffusion coefﬁcient, Dfast= pseudo-diffusion coefficient, FP= pseudo-diffusion factor given as a percentage, P5th= 5th percentile, and P95th=95th percentile.

a=10^-6^mm^2^/s, b=10^-4^mm^2^/s.

Intestinal type (n=32), Mixed-type (n=24), Diffuse-type (n=24).

**Table E3 Univariate analyses of IVIM parameters and ADC histogram/texture metrics between SRCs and other poorly cohesive adenocarcinomas**

| **Metrics** | **Median** | **P5th** | **P95th** | **Skewness** | **Kurtosis** | **Entropy** |
| --- | --- | --- | --- | --- | --- | --- |
| **Parameters** |  |  |  |  |  |  |
| **ADC** |  |  |  |  |  |  |
| Other PCs | 1450.71^a^ | 801.79^a^ | 2269.79^a^ | 0.37 | 0.45 | 2.94 |
| 95% CI | [1293.36-1608.07] | [636.37-967.20] | [2096.85-2442.72] | [0.13-0.61] | [0.69-0.84] | [2.83-3.06] |
| SRCs | 1265.67^a^ | 672.50^a^ | 2094.66^a^ | 0.37 | 0.55 | 2.91 |
| 95% CI | [1164.42-1366.91] | [488.85-856.15] | [1957.56-2231.77] | [0.07-0.67] | [0.07-0.68] | [2.82-3.00] |
| P value | 0.05 | 0.265 | 0.105 | 0.991 | 0.823 | 0.626 |
| **Dslow** |  |  |  |  |  |  |
| Other PCs | 1197.00^a^ | 575.21^a^ | 1943.25^a^ | 0.44 | 0.88 | 3.06 |
| 95% CI | [1046.52-1347.48] | [410.79-739.64] | [1856.50-2370.50] | [0.15-0.73] | [0.39-1.37] | [2.97-3.16] |
| SRCs | 1020.00^a^ | 630.50^a^ | 1944.92^a^ | 0.37 | 0.69 | 2.94 |
| 95% CI | [960.50-1072.25] | [534.50-667.50] | [1849.77-2040.06] | [0.09-0.65] | [0.35-1.02] | [2.91-3.06] |
| P value | 0.041 | 0.742 | 0.374 | 0.698 | 0.498 | 0.297 |
| **Dfast** |  |  |  |  |  |  |
| Other PCs | 134.64^b^ | 15.68^b^ | 487.43^b^ | 1.55 | 3.24 | 1.94 |
| 95% CI | [107.56-161.72] | [13.66-17.70] | [405.79-569.06] | [1.25-1.85] | [0.77-4.36] | [1.73-2.14] |
| SRCs | 109.00^b^ | 14.50^b^ | 408.58^b^ | 1.53 | 4.51 | 1.97 |
| 95% CI | [85.50-132.50] | [12.50-16.50] | [332.27-484.89] | [1.08-1.98] | [1.63-7.39] | [1.77-2.16] |
| P value | 0.297 | 0.432 | 0.142 | 0.936 | 0.899 | 0.831 |
| **FP** |  |  |  |  |  |  |
| Other PCs | 21.23% | 2.75% | 53.13% | 0.90 | 0.33 | 2.56 |
| 95% CI | [16.18%-26.28%] | [2.35%-3.65%] | [47.23%-59.03%] | [0.55-1.25] | [-0.42 to 1.44] | [2.34-2.78] |
| SRCs | 19.57% | 3.12% | 52.51% | 0.96 | 0.56 | 2.59 |
| 95% CI | [15.44%-23.70%] | [2.31%-3.92%] | [46.31%-58.70%] | [0.64-1.28] | [-0.37-1.61] | [2.41-2.76] |
| P value | 0.593 | 0.781 | 0.876 | 0.787 | 0.781 | 0.841 |

Data from normal distribution shown as mean, Data from non-normal distribution shown as median.

Numbers in brackets are 95% confidence intervals (95% CI).

SRCs= Signet-ring cell carcinomas, PCs= poorly cohesive adenocarcinomas, ADC= apparent diffusion coefficient, Dslow= pure molecular-based diffusion coefﬁcient, Dfast= pseudo-diffusion coefficient, FP= pseudo-diffusion factor given as a percentage, P5th= 5th percentile, and P95th= 95th percentile.

a=10^-6^mm^2^/s, b=10^-4^mm^2^/s.

Other PCs (n=14), SRCs (n=12).
